# MicroRNA analysis reveals the role of miR-214 in duck adipocyte differentiation

**DOI:** 10.5713/ab.21.0441

**Published:** 2022-01-21

**Authors:** Laidi Wang, Xiaodan Hu, Shasha Wang, Chunyou Yuan, Zhixiu Wang, Guobin Chang, Guohong Chen

**Affiliations:** 1 Key Laboratory of Animal Genetics and Breeding and Molecular Design of Jiangsu Province, Yangzhou University, Yangzhou 225009, China

**Keywords:** Adipocyte, *CPT2*, Differentiation, Duck, miR-214

## Abstract

**Objective:**

Fat deposition in poultry is an important factor in production performance and meat quality research. miRNAs also play important roles in regulating adipocyte differentiation process. This study was to investigate the expression patterns of miRNAs in duck adipocytes after differentiation and explore the role of miR-214 in regulating carnitine palmitoyltransferases 2 (*CPT2*) gene expression during duck adipocyte differentiation.

**Methods:**

Successful systems for the isolation, culture, and induction of duck primary fat cells was developed in the experiment. Using Illumina next-generation sequencing, the miRNAs libraries of duck adipocytes were established. miRanda was used to predict differentially expressed (DE) miRNAs and their target genes. The expression patterns of miR-214 and *CPT2* during the differentiation were verified by quantitative real-time polymerase chain reaction and western blot. Luciferase reporter assays were used to explore the specific regions of *CPT2* targeted by miR-214. We used a miR-214 over-expression strategy *in vitro* to further investigate its effect on differentiation process and *CPT2* gene transcription.

**Results:**

There were 481 miRNAs identified in duck adipocytes, included 57 DE miRNA candidates. And the 1,046 targets genes of DE miRNAs were mainly involved in p53 signaling, FoxO signaling, and fatty acid metabolism pathways. miR-214 and *CPT2* showed contrasting expression patterns before and after differentiation, and they were selected for further research. The expression of miR-214 was decreased during the first 3 days of duck adipocytes differentiation, and then increased, while the expression of *CPT2* increased both in the transcriptional and protein level. The luciferase assay suggested that miR-214 targets the 3′untranslated region of *CPT2*. Overexpression of miR-214 not only promoted the formation of lipid droplets but also decreased the protein abundance of *CPT2*.

**Conclusion:**

Current study reports the expression profile of miRNAs in duck adipocytes differentiated for 4 days. And miR-214 has been proved to have the regulator potential for fat deposition in duck.

## INTRODUCTION

Deposited fat in poultry mainly consists of abdominal fat, subcutaneous fat, and intramuscular fat (IMF). Excessive fat deposition can reduce feed efficiency and carcass yield [1]. While the IMF is recognized as the predominant factor affecting meat quality due to its positive correlation with juiciness, tenderness, and flavor. Therefore, understanding fat deposition is critical in meat quality research and improving production [2]. The molecular and cellular mechanisms of underlying lipid metabolism have been well studied in mammalian models; for instance, the increasing demands of muscle during exercise or heat production depend on fat mobilization [3], and fatty acid oxidation is essential for constraining adipocyte lipolysis and regulating systemic catabolism when glucose is limiting [4]. In contrast, such information is scarce for birds.

Adipocytes, which are derived from mesenchymal stem cells, play an important role in energy balance in both poultry and mammals [5]. Adipose tissue expansion is a result of adipocyte formation, involving several processes collectively referred to as adipogenesis, and cellular accumulation of triglyceride (TG) inside lipid droplets [6]. For the last 40 years, the cellular and molecular mechanisms of adipocyte differentiation have been extensively studied using pre-adipocyte culture systems [7]; specifically, committed pre-adipocytes undergo growth arrest and subsequent terminal differentiation into adipocytes. Similar vitro differentiation system has also been widely used in the adipocyte differentiation researches in both chickens and ducks [8,9].

MicroRNA (miRNA) is a class of noncoding RNA, with 18 to 25 nucleotides (nt) in length, which commonly acted as the negative regulators of gene expression at post-transcriptional levels [10]. Numerous miRNAs are present in adipocytes [11]. For instance, miRNA-18b-3p [12], miRNA-223 [13], miRNA-15a [14], and miR-21 [15] regulate adipocyte differentiation in chickens, by binding the 3′ untranslated region (UTR) of their target mRNAs. And miR-214 is related to glucose and lipid metabolism [16]. miR-214 is proved to target catenin beta 1 (*CTNNB1*) to promote differentiation by interfering with the Wnt/β-Catenin signaling pathway, in 3T3-L1 preadipocytes [17], duck adipocytes [18] and periodontal ligament stem cells [19]. While the mechanism of miR-214 in adipocyte differentiation has not been fully revealed.

In this study, high-throughput sequencing technology was used to monitor miRNA levels in duck embryo primary preadipocytes and differentiated adipocytes. miR-214 and *CPT2* were selected for further analysis at different stages of differentiation. Overexpression of miR-214 promoted adipocytes differentiation and downregulated the protein level of *CPT2*. Collectively, these results not only expand the miRNA regulation network during adipogenesis but also provide potential molecular regulator for fat deposition in duck.

## MATERIALS AND METHODS

### Ethics statement

All animal experimental procedures were approved and guided by the Institutional Animal Care and Use Committee of Yangzhou University (approval number 176–2020).

### Duck pre-adipocyte isolation, culture, and differentiation

Primary pre-adipocytes were isolated from subcutaneous adipose tissue of twenty-day-old embryonic Cherry Valley ducks, using the method established previously [18]. The pre-adipocytes were seeded at a density of 1×10^4^ cells/cm^2^ and maintained in complete medium (Dulbecco’s modified eagle medium with 10% fetal bovine serum, and 1% penicillin and streptomycin) [20]. Upon reaching 90% cell confluence, the medium was replaced with the differentiation medium, comprising the complete medium with an additional 300 μM oleic acid, 1 μm/L dexamethasone, 0.5 mmol/L 3-isobutyl-1-methylxanthine, 1 μM rosiglitazone, 10 μg/μL insulin, added. The medium was replaced every second day [8]. Pre-adipocyte morphology was observed using a microscope (Olympus, Tokyo, Japan).

### Oil red O staining and triglyceride content analysis

Oil red O staining kit (Solarbio, Beijing, China) were used for the lipid droplets staining. Firstly, adipocytes were washed with phosphate-buffered saline (PBS) and fixed using 4% formaldehyde for 10 min. Then, stained the cells with 1% filtered oil red O solution after two rinses in PBS. After 30 min, the cells were repeatedly washed with distilled water and observed under a microscope. For oil red O quantitative analysis, the intracellular adsorbed oil red O was extracted in 100% isopropanol for 10 min, and absorbance was measured at 500 nm wavelength.

Triglyceride content was analyzed in three replicates using a TG detection kit (Applygen, Beijing, China) according to the manufacturer’s protocols. Briefly, duck adipocytes were washed using preheated PBS and then centrifuged at 1,000 ×*g* for 5 min. The cells were ruptured using the ultrasonic cell-break method. A bicinchoninic acid protein assay kit (Beyotime, Beijing, China) was used to measure the protein concentration of the ruptured cells. Optical density values were detected using a microplate spectrophotometer.

### Small RNAs library construction and RNA-seq analysis

Three Cherry Valley duck pre-adipocytes samples (CVC) and those three samples that had undergone differentiation treatment for 3 d (CVT) were collected for RNA sequencing using the mirVana miRNA Isolation Kit (Ambion, Austin, TX, USA). After quality checking, total RNA for each sample was used to construct the small RNA library, according to the manufacturer’s protocol, using the Illumina Small RNA Sample Prep Kit (Illumina, San Diego, CA, USA). Libraries were sequenced using the Illumina HiSeq 2500 platform by paired-end sequencing, conducted by Novogene Biotechnology Co., Ltd. (Beijing, China).

### Identification of miRNA and differentially expressed miRNAs

After conducting quality control, the clean reads for each sample were mapped using Bowtie [21] against the standard duck genome BGI_duck_1.0 (https://www.ncbi.nlm.nih.gov/assembly/GCA_000355885.1), then aligned with the known miRNAs in miRbase (https://www.mirbase.org/index.shtml) to identify known miRNAs, allowing less than one mismatch between sequences. The identified miRNAs were classified into families based on their sequence similarities. Unmatched reads were further processed to predict novel miRNAs using miREvo [22] and mirdeep2 [23]. Differentially expressed (DE) miRNA (read count threshold >10) analysis was performed using the DEseq 2 algorithm, with the cutoff of |Log_2_ Fold Change|>1 and false discovery rate <0.05. Six miRNAs (miR-10b-3p, miR-455-5p, miR-148a-3p, miR-122-5p, miR-15c-5p, and miR-10a-5p) were randomly selected for validation using a replicated experiment. The quantitative real-time polymerase chain reaction (qRT-PCR) primer sequences used are listed in Table 1.

### Prediction of miRNA target genes and pathway enrichment analysis

Differentially expressed miRNAs and their target genes were predicted using miRanda (https://www.microrna.org/microrna/home.do) based on the previous transcriptome results [8]. Gene ontology (GO) and Kyoto encyclopedia of genes and genomes (KEGG) enrichment analyses were performed using DAVID 6.8 (https://david.ncifcrf.gov/). Less than 0.05 p value was considered significantly enriched. Signal pathway diagram of target genes involved in fatty acid metabolism was performed by Microsoft PowerPoint.

### Sample collection and quantitative real-time polymerase chain reaction

Three seven-day-old male Cherry Valley ducks were euthanized by cervical dislocation, and the heart, liver, spleen, lung, breast muscle, right leg muscle, and adipose tissues were collected to analyze gene expression. Total RNA was extracted from the tissue or cultured cells using Trizol reagent (Invitrogen, Carlsbad, CA, USA). First-strand cDNA was synthesized from 1 μg of total RNA using the PrimeScript RT-PCR Kit (TaKaRa, Osaka, Japan), following the manufacturer’s instructions. The mRNA expression levels of several marker genes were detected using a TB Green qRT-PCR Premix (TaKaRa, Japan) and the QuantStudio 5 real-time PCR instrument. Mir-X miRNA First-Strand Synthesis (TaKaRa, Japan) and TB Green qRT-PCR Premix (TaKaRa, Japan) were used for qRT-PCR analysis of miR-214. The qRT-PCR primer sequences used are listed in Table 1 and Table 2, while the common downstream primers for miRNA were provided by Takara (undisclosed sequence). Beta-actin or *U6* were used as internal controls, and all assays were run in triplicate. Relative transcription was evaluated using the 2^−ΔΔCt^ method, and is presented as mean±standard deviation.

### Vector construction and luciferase assays

A luciferase reporter, with two wild type target sites of the *CPT2* 3′ UTR (WT), was constructed by subcloning the fragment into the region directly downstream of the cytomegalovirus promoter-driven firefly luciferase cassette in the pMIR-report vector (Promega, Madison, WI, USA). Three mutations of the miR-214-binding site were synthesized—at the first binding site (MUT1), second binding site (MUT2), or at both binding sites (MUT3)—by Sangon Co., Ltd., Shanghai, China. The vector construction primer sequences are shown in Table 2. The constructs were verified by Sanger sequencing. The miR-214 mimics (5′-GACGGACAGACAC GGACGACA-3′) and miRNA negative controls (5′-UCAC AACCUCCUAGAAAGAGUAGA-3′) used for cell transfection were synthesized by Ribobio Co., Ltd., Guangzhou, China. For the resulting pre-adipocytes, 50 nM of miRNA was transfected into the pre-adipocytes using Lipofectamine 2000 Reagent (Life Technologies, Grand Island, NY, USA). Firstly, dilute three amounts of Lipofectamine 2000 Reagent or miR-214 mimics in Opti-MEM Medium respectively. Then, add diluted miR-214 mimics to diluted Lipofectamine 2000 Reagent. After 15 minutes incubation at room temperature, DNA-lipid complex were added to cells. Differentiation medium were added for three days after 48 h post-transfection.

To validate the miRNA targets, we used 293T cells provided by Professor Song (Yangzhou University, China). Cells were seeded into 24-well plates at a density of 1×10^5^ cells/well, and incubated at 37°C overnight. Co-transfection with 100 ng of the WT or MUT vectors, and with 2 μL of the miR-214 mimics or negative control, were performed using Lipofectamine 2000 (Invitrogen, USA). Luciferase activity was measured using the Dual Luciferase Reporter Assay System (Promega, USA) at 48 h post-transfection. Finally, the foldchange in expression was calculated by comparing the expression of miR-214 with that of the miRNA-NC.

### Western blot analysis

Total protein was extracted from cells using RIPA buffer (Beyotime, Shanghai, China) supplemented with a protease inhibitor cocktail (BioDee, Beijing, China) (100:1) and phenylmethylsulfonyl fluoride (Beyotime, Shanghai, China) (100:1). Proteins were separated on 8% sodium dodecyl sulfate-polyacrylamide gel electrophoresis gels (GenScript, Nanjing, China). The proteins were transferred to polyvinylidene difluoride membranes, and then blocked with 5% non-fat milk for 1 h. The membranes were incubated with the primary antibodies CPT2 (1:1,000, NBP1-32226; Novus, Columbia, USA) and beta-actin (1:5,000, NB600-532SS; Novus, USA) for 1 h, and washed with PBST (Solarbio, China) three times, for 5 min each time. The membranes were then incubated with anti-rabbit secondary antibody conjugated with horseradish peroxidase (1:1,500, 7074; Cell Signaling Technology, Boston, MA, USA) for 1 h at room temperature, and washed three times using PBST. Protein bands were visualized using an enhanced chemiluminescence system (GE Healthcare, Fairfield, CT, USA).

### Statistical analysis

Statistical analysis was performed using SPSS 22.0 and GraphPad Prism 6. Meaningful differences between the groups were calculated using Student’s *t*-tests. Statistical significance is defined when p values are less than 0.05.

## RESULTS

### Comparison of differentiated and non-differentiated preadipocytes *in vitro*

**Figure 1:**
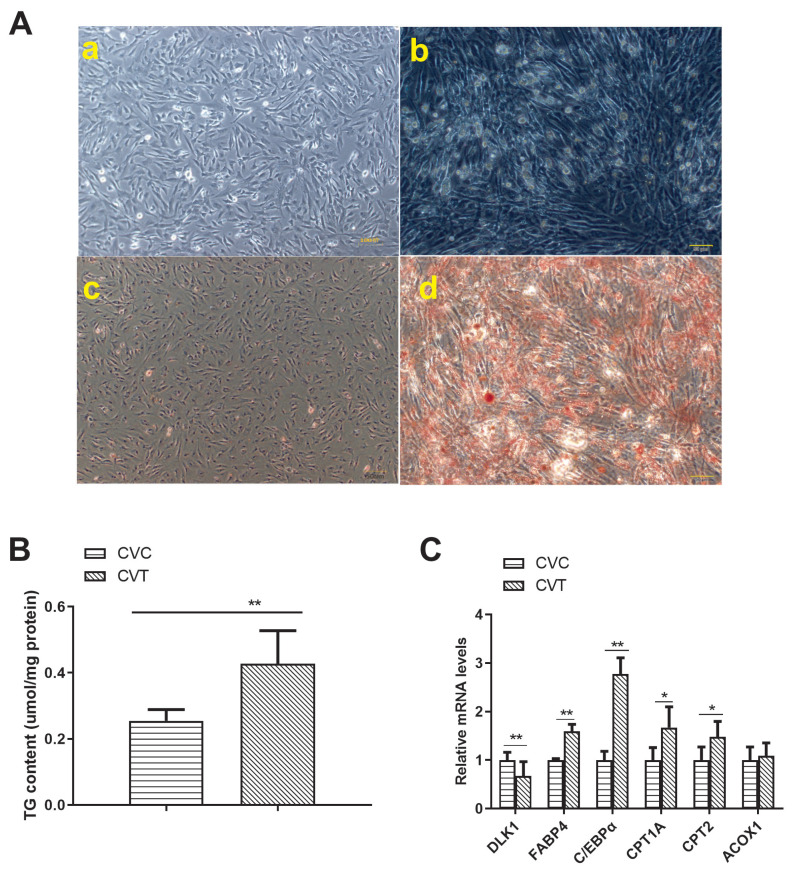
*In vitro* comparison of differentiated and non-differentiated Cherry Valley duck preadipocytes. (A) Comparison of (a) non-differentiated Cherry Valley duck preadipocytes (CVC) and (b) Cherry Valley duck adipocytes differentiated for 3 d (CVT); Oil Red O staining of (c) CVC (d) CVT cells. (B) Triglyceride (TG) content of CVC and CVT cells. Triglyceride content is expressed in mmol/g protein. (C) Relative mRNA expression of six genes related to differentiation and fatty acid metabolism in adipocytes. Beta-actin was used as an endogenous control for relative quantification. (B, C) Data are shown as the mean±standard deviation. n = 3, * p<0.05, ** p<0.01.

Oil red O staining revealed that there were accumulated lipid droplets in differentiated duck adipocytes (Figure 1A). Triglyceride content was significantly higher in the differentiated adipocytes than in the preadipocytes (Figure 1B). After differentiation, the genes related to lipid metabolism (delta-like non-canonical Notch ligand 1 [*DLK1*], CCAAT/enhancer-binding protein-α [*C/EBPα*], adipocyte fatty-acid binding protein 4 [*FABP4*], carnitine palmitoyltransferases 1A [*CPT1A*], CPT2, and acyl-coenzyme A oxidase 1 [*ACOX1*]), were detected in the adipocyte cells (Figure 1C). The expression of *C/EBPα*, *FABP4*, *CPT1A*, and *CPT2* was higher, and that of *DLK1* was lower, in differentiated adipocytes than in pre-adipocytes. The differentiated adipocytes showed normal differentiation progress and higher fatty acid transport.

### Summary of small RNA-seq data

In total, 12,244,755, 10,749,053, 11,688,816, 11,178,383, 11,706,221, and 11,456,503 raw reads were obtained from the CVC1, CVC2, CVC3, CVT1, CVT2, and CVT3 libraries, respectively (Supplementary Table S1). After removing contaminant reads, we obtained 11,614,612, 10,227,841, and 11,095,066 clean reads for CVC, and 10,650,292, 11,006,776, and 10,615,983 clean reads for CVT, of 18 to 35 nt long, which were used for subsequent analyses. These clean reads were uniquely mapped to the standard duck genome BGI_duck_1.0 (https://www.ncbi.nlm.nih.gov/assembly/GCA_000355885.1); on average, 10,979,173 and 10,757,684 clean reads were mapped for the CVC and CVT libraries, respectively. Further, >90% of the bases had base quality >Q20. Most of the clean reads were 21 to 24 nt; 22 nt reads were the most abundant, followed by 23 nt reads (Supplementary Figure S2A). The sequenced sRNAs mapped to >60% of miRNAs in the miRBase database (Supplementary Table S2). However, the other types of RNAs (including rRNAs, tRNAs, mRNAs, and snoRNA) were mapped in relatively low proportions. Reproducibility between samples was good (Supplementary Figure S2B). This indicates that the deep sequencing data were highly enriched in mature miRNA sequences and were suitable for subsequent profiling analysis.

### Differentially expressed miRNAs between CVC and CVT cells

**Figure 2:**
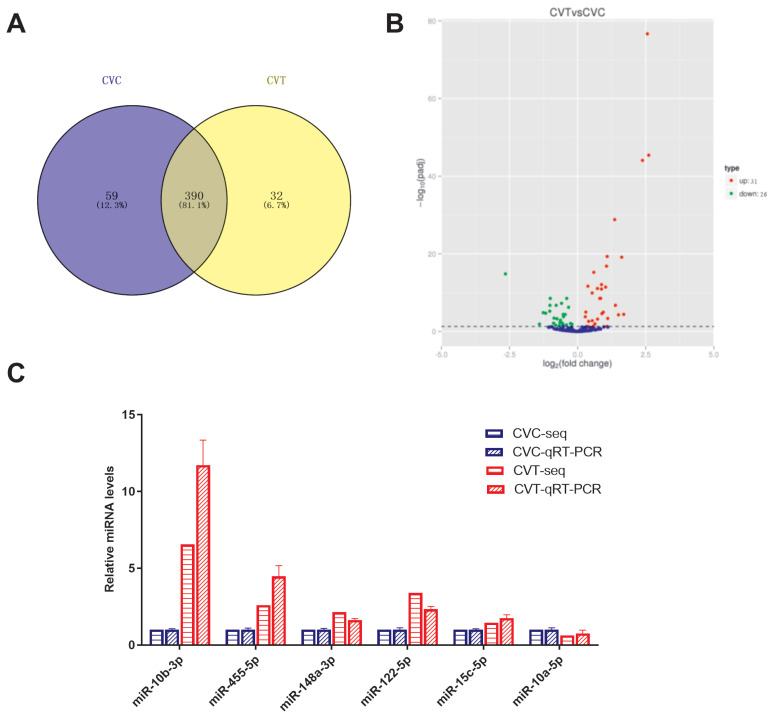
Analysis of differentially expressed miRNAs, comparing differentiated and non-differentiated Cherry Valley duck preadipocytes (CVT and CVC cells, respectively). (A) Venn diagrams showing the numbers of common and unique miRNAs, comparing CVC and CVT cells. (B) Volcano plot of differentially expressed miRNAs, comparing CVC and CVT cells. (C) quantitative real-time polymerase chain reaction (qRT-PCR) verification of sequencing results.

To identify miRNAs that may play key regulatory roles in abdominal fat production, 481 miRNAs were identified in the CVC and CVT cells; of these, 390 were common to both groups (Figure 2A). There were 57 DE miRNAs (Supplementary Table S3), included 31 upregulated miRNAs, and 26 downregulated miRNAs (Figure 2B). The three conserved miRNA families were differentially expressed (p<0.05): miR-10 (miR-10a-5p, -10b-3p, -10b-5p), miR-146 (miR-146b-3p, -146b-5p, -146c-5p), miR-30 (miR-30a-3p, -30a-5p,-30c-1-3p, -30d). To validate the miRNA-seq results, 6 miRNAs were randomly selected for qPCR assay; the qPCR relative expression levels were consistent with the miRNA-Seq data (Figure 2C). This indicates that the miRNA identification and abundance estimation were reliable.

### Prediction and functional annotation of differentially expressed miRNA target genes

**Figure 3:**
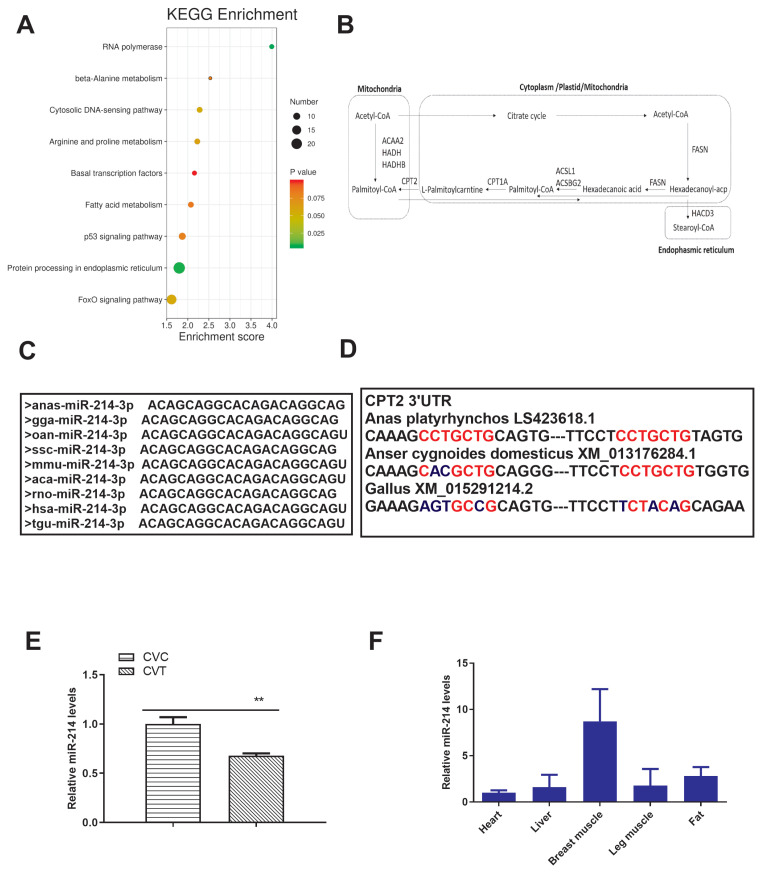
Functional enrichment analysis of differentially expressed miRNAs and their target genes, comparing differentiated and non-differentiated Cherry Valley duck preadipocytes (CVT and CVC cells, respectively). (A) KEGG analysis of the targets of differentially expressed miRNAs. (B) Signal pathway diagram of target genes involved in fatty acid metabolism. (C) Conservatism analysis of the miR-214 seed region. (D) Conservatism analysis of the target sites of miR-214 in the 3′ UTR of *CPT2*. (E) qRT-PCR verification of miR-214 expression. (F) Expression of miR-214 in various duck tissues, detected using qPCR. U6 small nuclear RNA (snRNA) was used as an endogenous control for miR-214 relative quantification. Data are shown as the mean±standard deviation from three individuals. KEGG, Kyoto encyclopedia of genes and genomes; UTR, untranslated region; *CPT2*, carnitine palmitoyltransferases 2; qRT-PCR, quantitative real-time polymerase chain reaction. ** p<0.01.

In total, 1,046 target genes were associated with the 57 differentially expressed miRNAs (p<0.05). KEGG pathway enrichment revealed that the target genes were enriched in the p53 signaling pathway, protein processing in endoplasmic reticulum, and the FoxO signaling pathway (Figure 3A); further, 3-hydroxyacyl-CoA dehydrogenase β (*HADHB*), acetyl CoA acyltransferase 2 (*ACAA2*), *CPT2*, acyl-CoA synthetase 1 (*ACSL1*), fatty acid synthase (*FASN*), hydroxyacyl-CoA dehydrogenase (*HADH*), hydroxyacyl-CoA dehydratase 3 (*HACD3*), and acyl-CoA synthetase bubblegum family member 2 (*ACSBG2*) were associated with fatty acid metabolism (Figure 3B, Supplementary Table S4). Interestingly, there were two target sites of miR-214 in the 3′UTR of *CPT2* (Supplementary Table S5, Supplementary Figure S1). Therefore, miR-214 and the target gene *CPT2* were selected for further research.

### miR-214 and *CPT2* expression in preadipocytes and adipocytes

The seed region of the mature miR-214 sequence was highly conserved in duck, chickens, mice, humans, and other species (Figure 3C). Two potential binding sites for miR-214 were identified in the 3′ UTR of the *CPT2* gene; these sites were not highly conserved among poultry (Figure 3D). There was a negative association between miR-214 expression and *CPT2* gene expression by the transcriptome results. We verified miR-214 expression levels in CVC and CVT cells (Figure 3E), and found that it was expressed in the heart, liver, breast muscle, thigh muscle, and fat tissue of 7 day-old Cherry Valley ducks (Figure 3F), but mostly in the breast muscle and adipose tissue.

**Figure 4:**
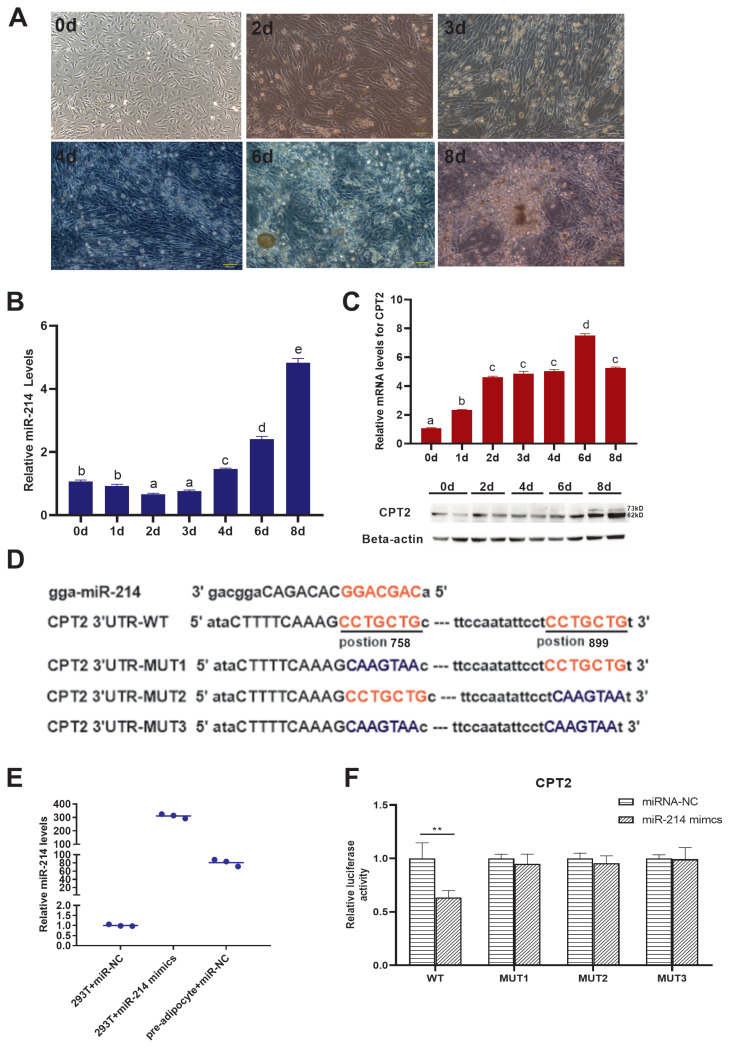
The relationship between *CPT2* and miR-214. (A) Changes in adipocyte morphology with different differentiation. Relative expression of (B) miR-214 and (C) CPT2 during differentiation. CPT2-73kD and CPT2-62kD: two variant proteins for duck CPT2. (D) Predicted binding and mutation sites in miR-214 and in the CPT2 3′ UTR. (E) miR-214 expression level in 293T and duck preadipocyte. (F) Luciferase assay verification of the miR-214 target CPT2 3′ UTR. CPT2, carnitine palmitoyltransferases 2; UTR, untranslated region. ^a–e^ Different letters within the same graph indicate significant difference p<0.05. ** p<0.01.

To fully elucidate miR-214 and *CPT2* expression during adipocyte differentiation, we assessed their expression at 0, 2, 4, 6, and 8 d post-differentiation. Lipids gradually accumulated to form larger lipid droplets as the differentiation time increased (Figure 4A). The expression level of miR-214 was decreased in the first 3d differentiation time and then increased rapidly (Figure 4B). The adipocytes differentiation induced the expression of CPT2 at the transcriptional level and accumulation of the protein level (Figure 4C).

### miR-214 overexpression promoted adipocyte differentiation and targeting *CPT2*

In order to verify the miR-214 target sites on *CPT2*, a plasmid containing the WT or mutant target sites of the 3′ UTR region (Figure 4D) of the *CPT2* gene was constructed. Besides, miR-214 expression level in 293T was lower than that in duck preadipocyte, and the effect of miR-214 mimics overexpression could meet the experimental requirements (Figure 4E). The plasmid were transfected into 293T cells, together with miR-214 mimics or miR-NC. miR-214 mimics significantly reduced the luciferase activity of the WT *CPT2* 3′ UTR, but did not affect the activity of the three *CPT2*-3′ UTR mutants (Figure 4F). This indicates that miR-214 binds directly to the two target sites in the *CPT2* 3′ UTR, and that there might be a cascade effect between the two target sites.

**Figure 5:**
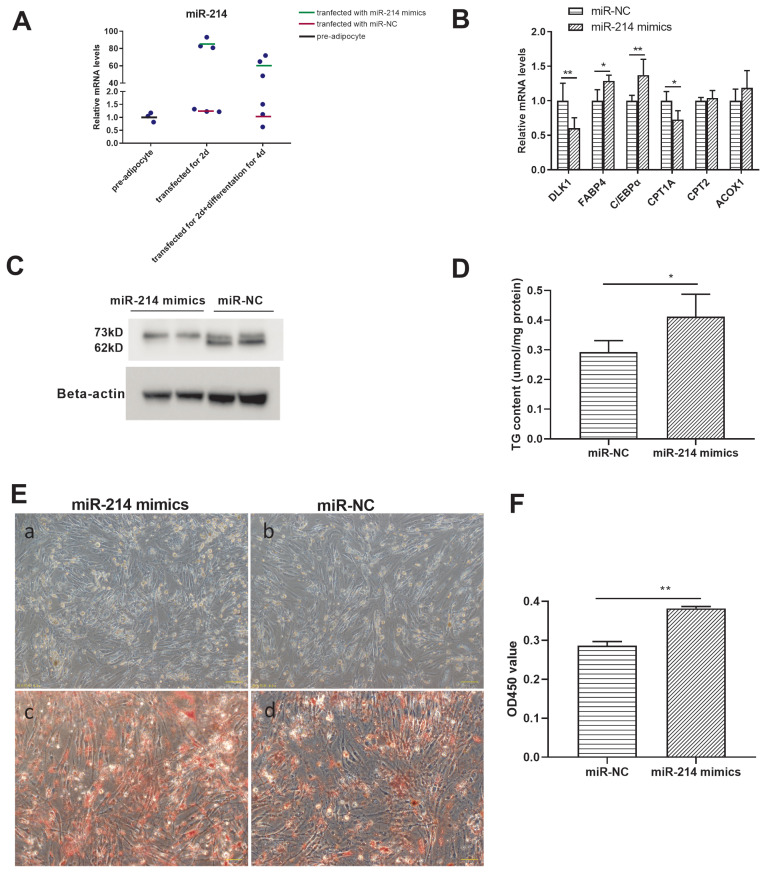
The targeting effect of miR-214 and CPT2 in Cherry Valley duck adipocyte differentiation. (A) Efficacy of miR-214 mimics. (B) Gene expression and (C) CPT2 protein expression, in pre-adipocytes treated with the miR-214-mimic or the negative control (NC). (D) Triglyceride content of adipocytes transfected for 48 h with miR-214 mimics or with the miR-NC, and following differentiation for 4 d. (E) Comparison of preadipocytes transfected with miR-NC (a) or with miR-214 mimics (b) for 48 h, and following differentiation for 4 d. Oil red O staining of adipocytes transfected with miR-NC (c) or with miR-214 mimics (d) for 48 h, and following differentiation for 4 d. (F) Spectrophotometric analysis following oil red O staining. CPT2, carnitine palmitoyltransferases 2. * p<0.05, ** p<0.01.

To investigate the regulatory role of miR-214 in adipocyte differentiation, an overexpression experiment was conducted, by transfecting miR-214 mimics. miR-214 expression was significantly higher in the overexpression groups (those transfected with the miR-214 mimics), for both preadipocytes and differentiated adipocytes, than in the groups transfected with the miR-NC (Figure 5A). *FABP4*, *C/EBPα*, and *CPT1A* expression was higher, and that of *DLK1* was lower, in the miR-214 over-expression group after differentiation, than in the negative control group (Figure 5B). miR-214 over expression inhibited the expression level of CPT2-73kD protein but not at the level of mRNA (Figure 5C). Besides, triglyceride content was higher in the overexpression group than in the negative control group (Figure 5D). The number of lipid droplets was increased and the lipid droplets become bigger after miR-214 overexpression (Figures 5E, F; Supplementary Figure S2). Together, these results suggested that miR-214 overexpression promoted adipocyte differentiation process, reduced fatty acid transfer to mitochondria.

## DISCUSSION

In this study, we found that, after 4d of culture in differentiation medium, TG content increased, and lipid droplets gradually accumulated inside cells. The expression level of fatty acid synthesis genes increased, and that of fatty acid transport genes increased slightly. And, several of the miRNAs identified in our miRNA-seq analysis, such as miR-103, miR-21, miR-30, miR-27, miR-130 and let-7, were also detected by other researches [11,24]. Further, these miRNAs are known to be associated with adipocyte differentiation; in porcine preadipocytes, for instance, miR-130a overexpression can inhibit adipocyte differentiation [25]; in Zebrafish, miR-27b depletion promotes lipid accumulation and weight gain [26], and in chicken, inhibiting miR-30a promotes adipocyte differentiation [27]. Our study revealed differential miRNA expression related to adipocyte differentiation.

We conducted KEGG pathway analysis to reveal the biological significance of the target genes of the DE miRNAs. The “fatty acid metabolism” pathway was enriched in KEGG analysis and 8 genes (*HADHB*, *ACAA2*, *CPT2*, *ACSL1*, *FASN*, *HADH*, *HACD3*, and *ACSBG2*) were involved in this pathway. They play important roles in fatty acid synthesis, transport, and oxidative utilization. *FASN* and *HACD3* are two key enzymes required for fatty acid synthesis [28,29]. *ACSL1* and *ACSBG2* enable fatty acids to enter into lipid pools, and activate fatty acids [30,31]. *CPT1* and *CPT2* mediate fatty acid transfer into the mitochondrial matrix for β-oxidation [32]. And the study of Qiu proved that the expression level of *CPT1* regulated fatty acid oxidation and thus affects intramuscular adipogenesis [33]. *CPT2* was proved to be a common up-regulated protein during differentiation of preadipocytes from bovine omental, subcutaneous and intramuscular adipose depots [34]. *HADH*, *HADHB*, and *ACAA2* are key enzymes in fatty acid oxidation [35–37]. Some of these enzymes have been shown to be regulated by miRNAs; for instance, miR-33 regulates fatty acid metabolism by targeting *HADHB*, in chickens [38], geese [39], and African green monkeys [40]; and miR-214 is predicted to regulate *ACSBG2* expression [27]. We suggest that the transport and utilization of fat is closely related to the accumulation of fat droplets. Considering that two target sites of miR-214 in the 3′UTR of *CPT2*, miR-214 and *CPT2* are selected to explore their regulatory mechanism during adipocyte differentiation in duck.

miR-214 is an important miRNA that has been well studied in humans. It is also identified in chicken [27], duck [18], and pigeon [41]. Although, miR-214 is a highly conserved miRNA between humans and the poultry above, its function in adipocyte differentiation and fatty acid metabolism in ducks has not been fully reported. It has multiple functions in both normal and cancer cells, helps to coordinate essential signaling pathways [42]. It is also a potential biomarker or target for therapeutic intervention [43]. Besides, miR-214 is implicated in promoting fibroblast differentiation of adipose-derived mesenchymal stem cells by targeting mitofusin-2 (*MFN2*) [44], and suppressing the osteogenic differentiation of bone marrow-derived mesenchymal stem cells and human hair follicle stem cells [45,46]. We found that the expression level of miR-214 was decreased in the early adipocyte differentiation, then increased in the later adipocyte differentiation. The research on 3T3-L1 preadipocyte differentiation revealed that the level of miR-214 down-regulated on 36h of differentiation and up-regulated on 6d of differentiation [17]. Further, miR-214 overexpression promoted duck adipocyte lipid droplets accumulation, this is consistent with findings for 3T3-L1 preadipocytes [17]. Taken together, our findings provided that miR-214 may be an important positive regulator in duck adipogenic development.

The carnitine palmitoyltransferase (*CPT*) enzyme system, which operates throughout the body, enables long-chain fatty acids to enter the mitochondrial matrix [47]. The *CPT* system comprises two separate proteins located in the outer and inner mitochondrial membranes (*CPT1* and *CPT2*, respectively) [32]. Carnitine is important for mitochondrial metabolism; however, two carnitine acyltransferases, carnitine octanoyltransferase and carnitine acetyltransferase, are peroxisomal enzymes, indicating that it is also important for peroxisomal metabolism. There is some evidence that peroxisomes can degrade fatty acids that are typically degraded by mitochondria, possibly after transport as acylcarnitines [47]. During early adipocyte differentiation, the lipid droplets accumulated and *CPT2* expression increased with differentiation time which is coincide with the result of bovine during differentiation of adipocytes [34]. Here, we investigated that, during the differentiation time, miR-214 and CPT2 did not show obvious opposite expression patterns, these might explained by the complex regulatory systems during adipocyte differentiation. Furthermore, *CPT2* regulated fatty acid transportation and differentiation in duck adipocytes: lipid droplets accumulated and *CPT2* expression increased with differentiation time, enhancing fatty acid transport; while the later up-regulated miR-214 promoted the differentiation process, inhibited the increasing expression of *CPT2*, and these further increased the accumulation of lipid droplets. *CPT2* is also found to be under the expression regulation of peroxisome proliferator-activated receptor γ (*PPARγ*) [34]. Besides, miR-214 overexpression enhanced duck adipocyte lipid droplets accumulation and reduced the protein level of *CPT2*, thereby reducing fatty acid transport and oxidation. Altogether, we suggest that miR-214 and *CPT2* are involved in the complex regulatory network during the duck adipocytes differentiation.

## Figures and Tables

**Table 1:** Primer sequences for miRNA quantification

Primers	Sequence (5′–3′)
miR-10b-3p-F	AGATTCGATTCTAGGGGAATA
miR-455-5p-F	TATGTGCCCTTGGACTACATCG
miR-148a-3p-F	TCAGTGCACTACAGAACTTTGT
miR-122-5p-F	TGGAGTGTGACAATGGTGTTTGT
miR-15c-5p-F	TAGCAGCACATCATGGTTTGTA
miR-10a-5p-F	TACCCTGTAGATCCGAATTTGT
miR-214-F	ACAGCAGGCACAGACAGGCAG
U6-F	TGGAACGCTTCACGAATTTGCG
U6-R	GGAACGATACAGAGAAGATTAGC

**Table 2:** Primer sequences for polymerase chain reaction

Primers	Sequence (5′–3′)	Product length (bp)
*DLK1*-F	CTGTGCCCTTCTGGTTTTGC	192
*DLK1*-R	TCCATTCTCACATGGGCCAC	
*FABP4*-F	AATGGCTCACTGAAGCAGGT	143
*FABP4*-R	TGGCTTCTTCATGCCTTTTC	
*C/EBPα*-F	GTGCTTCATGGAGCAAGCCAA	191
*C/EBPα*-R	TGTCGATGGAGTGCTCGTTCT	
*CPT1A*-F	CCTGGTGGGCCACAAACTAT	236
*CPT1A*-R	GAGCGGAACAGTTGATCCCA	
*CPT2*-F	TATCCGTCCTGCCTCTATTC	166
*CPT2-*R	GAGATGTCGGTCAAATCCCT	
*ACOX1*-F	CTGTGGTGGGCATGGCTATT	207
*ACOX1*-R	TGAATGCGTTGCCTGGAGAG	
*Beta-actin*-F	ATGTCGCCCTGGATTTCG	165
*Beta-actin*-R	CACAGGACTCCATACCCAAGAA	
*CPT2*-sac1-F	cgagctc-AACCTGCAGTCACCAACTGT	341
*CPT2*-hind3-R	cccaagctt-TGCAGCCAAAGGCAAGTACA	

*DLK1*, delta-like non-canonical Notch ligand 1; *FABP4*, fatty-acid binding protein 4; *C/EBPα*, CCAAT/enhancer-binding protein-α; *CPT1A*, carnitine palmitoyltransferases 1A; *ACOX1*, acyl-coenzyme A oxidase 1.

## References

[1] Nematbakhsh S, Chong PP, Selamat J, Nordin N, Idris LH, Abdull Razis AF (2021). Molecular regulation of lipogenesis, adipogenesis and fat deposition in chicken. Genes (Basel).

[2] Ding SR, Li GS, Chen SR (2021). Comparison of carcass and meat quality traits between lean and fat Pekin ducks. Anim Biosci.

[3] Frayn KN, Arner P, Yki-Järvinen H (2006). Fatty acid metabolism in adipose tissue, muscle and liver in health and disease. Essays Biochem.

[4] Lee J, Choi J, Scafidi S, Wolfgang MJ (2016). Hepatic fatty acid oxidation restrains systemic catabolism during starvation. Cell Rep.

[5] Koutnikova H, Auwerx J (2001). Regulation of adipocyte differentiation. Ann Med.

[6] Wang G, Kim WK, Cline MA, Gilbert ER (2017). Factors affecting adipose tissue development in chickens: a review. Poult Sci.

[7] Gregoire FM, Smas CM, Sul HS (1998). Understanding adipocyte differentiation. Physiol Rev.

[8] Wang SS, Zhang Y, Xu Q (2018). The differentiation of preadipocytes and gene expression related to adipogenesis in ducks (Anas platyrhynchos). PLoS One.

[9] Shang ZC, Guo L, Wang N, Shi H, Wang YX, Li H (2014). Oleate promotes differentiation of chicken primary preadipocytes in vitro. Biosci Rep.

[10] Shi CM, Huang FY, Gu XM (2016). Adipogenic miRNA and meta-signature miRNAs involved in human adipocyte differentiation and obesity. Oncotarget.

[11] Zhang R, Wang D, Xia ZY (2013). The role of microRNAs in adipocyte differentiation. Front Med.

[12] Sun GR, Li F, Ma XF (2019). gga-miRNA-18b-3p inhibits intramuscular adipocytes differentiation in chicken by targeting the ACOT13 gene. Cells.

[13] Li F, Li DH, Zhang M (2019). miRNA-223 targets the GPAM gene and regulates the differentiation of intramuscular adipocytes. Gene.

[14] Li GX, Fu SY, Chen Y (2019). MicroRNA-15a regulates the differentiation of intramuscular preadipocytes by targeting ACAA1, ACOX1 and SCP2 in chickens. Int J Mol Sci.

[15] Wang WS, Cheng M, Qiao SP, Wang YX, Li H, Wang N (2017). Gga-miR-21 inhibits chicken pre-adipocyte proliferation in part by down-regulating Kruppel-like factor 5. Poult Sci.

[16] Cheng F, Yuan G, He J, Shao Y, Zhang J, Guo X (2020). Aberrant expression of miR-214 is associated with obesity-induced insulin resistance as a biomarker and therapeutic. Diagn Pathol.

[17] Xi FX, Wei CS, Xu YT (2019). MicroRNA-214-3p targeting Ctnnb1 promotes 3T3-L1 preadipocyte differentiation by interfering with the Wnt/β-Catenin signaling pathway. Int J Mol Sci.

[18] Wang LD, Liang WS, Wang SS (2020). Circular RNA expression profiling reveals that circ-PLXNA1 functions in duck adipocyte differentiation. PLoS One.

[19] Cao FD, Zhan JL, Chen XF, Zhang K, Lai RF, Feng ZQ (2017). miR-214 promotes periodontal ligament stem cell osteoblastic differentiation by modulating Wnt/β-catenin signaling. Mol Med Rep.

[20] Ding F, Li QQ, Li L (2015). Isolation, culture and differentiation of duck (Anas platyrhynchos) preadipocytes. Cytotechnology.

[21] Langmead B, Trapnell C, Pop M, Salzberg SL (2009). Ultrafast and memory-efficient alignment of short DNA sequences to the human genome. Genome Biol.

[22] Wen M, Shen Y, Shi SH, Tang T (2012). miREvo: an integrative microRNA evolutionary analysis platform for next-generation sequencing experiments. BMC Bioinformatics.

[23] Friedländer MR, Mackowiak SD, Li N, Chen W, Rajewsky N (2012). miRDeep2 accurately identifies known and hundreds of novel microRNA genes in seven animal clades. Nucleic Acids Res.

[24] Zhang M, Li F, Ma XF (2019). Identification of differentially expressed genes and pathways between intramuscular and abdominal fat-derived preadipocyte differentiation of chickens in vitro. BMC Genomics.

[25] Wei W, Sun WX, Han HY, Chu WW, Zhang LF, Chen J (2017). miR-130a regulates differential lipid accumulation between intramuscular and subcutaneous adipose tissues of pigs via suppressing PPARG expression. Gene.

[26] Hsu CC, Lai CY, Lin CY, Yeh KY, Her GM (2018). MicroRNA-27b depletion enhances endotrophic and intravascular lipid accumulation and induces adipocyte hyperplasia in zebrafish. Int J Mol Sci.

[27] Ma XF, Sun JW, Zhu SP (2020). MiRNAs and mRNAs analysis during abdominal preadipocyte differentiation in chickens. Animals (Basel).

[28] Wu X, Qin L, Fako V, Zhang JT (2014). Molecular mechanisms of fatty acid synthase (FASN)-mediated resistance to anti-cancer treatments. Adv Biol Regul.

[29] Sawai M, Uchida Y, Ohno Y (2017). The 3-hydroxyacyl-CoA dehydratases HACD1 and HACD2 exhibit functional redundancy and are active in a wide range of fatty acid elongation pathways. J Biol Chem.

[30] Ellis JM, Frahm JL, Li LO, Coleman RA (2010). Acyl-coenzyme A synthetases in metabolic control. Curr Opin Lipidol.

[31] Lopes-Marques M, Machado AM, Ruivo R, Fonseca E, Carvalho E, Castro LFC (2018). Expansion, retention and loss in the Acyl-CoA synthetase “Bubblegum” (Acsbg) gene family in vertebrate history. Gene.

[32] Bonnefont JPD, Prip-Buus F, Gobin C, Munnich S, Bastin AJ (2004). Carnitine palmitoyltransferases 1 and 2: biochemical, molecular and medical aspects. Mol Aspects Med.

[33] Qiu F, Xie L, Ma JE (2017). Lower expression of SLC27A1 enhances intramuscular fat deposition in chicken via down-regulated fatty acid oxidation mediated by CPT1A. Front Physiol.

[34] Rajesh RV, Heo GN, Park MR (2010). Proteomic analysis of bovine omental, subcutaneous and intramuscular preadipocytes during in vitro adipogenic differentiation. Comp Biochem Physiol Part D Genomics Proteomics.

[35] Das AM, Illsinger S, Lücke T (2006). Isolated mitochondrial long-chain ketoacyl-CoA thiolase deficiency resulting from mutations in the HADHB gene. Clin Chem.

[36] Shen C, Song YH, Xie Y (2017). Downregulation of HADH promotes gastric cancer progression via Akt signaling pathway. Oncotarget.

[37] Zhang YN, Wang YJ, Wang XY (2019). Acetyl-coenzyme A acyltransferase 2 promote the differentiation of sheep precursor adipocytes into adipocytes. J Cell Biochem.

[38] Shao F, Wang XG, Yu JF, Shen K, Qi C, Gu ZL (2019). Expression of miR-33 from an SREBP2 intron inhibits the expression of the fatty acid oxidation-regulatory genes CROT and HADHB in chicken liver. Br Poult Sci.

[39] Zheng Y, Jiang SB, Zhang YH, Zhang R, Gong DQ (2015). Detection of miR-33 expression and the verification of its target genes in the fatty liver of geese. Int J Mol Sci.

[40] Rayner KJ, Esau CC, Hussain FN (2011). Inhibition of miR-33a/b in non-human primates raises plasma HDL and lowers VLDL triglycerides. Nature.

[41] Jiang L, Bi D, Ding H, Ren Q, Wang P, Kan X (2019). Identification and comparative profiling of gonadal microRNAs in the adult pigeon (Columba livia). Br Poult Sci.

[42] Penna E, Orso F, Taverna D (2015). miR-214 as a key hub that controls cancer networks: small player, multiple functions. J Invest Dermatol.

[43] Sharma T, Hamilton R, Mandal CC (2015). miR-214: a potential biomarker and therapeutic for different cancers. Future Oncol.

[44] Wu J, Li J, Chen WK (2017). MicroRNA-214 affects fibroblast differentiation of adipose-derived mesenchymal stem cells by targeting Mitofusin-2 during pelvic floor dysfunction in SD rats with birth trauma. Cell Physiol Biochem.

[45] Du KT, Deng JQ, He XG, Liu ZP, Peng C, Zhang MS (2018). MiR-214 regulates the human hair follicle stem cell proliferation and differentiation by targeting EZH2 and Wnt/β-Catenin signaling way in vitro. Tissue Eng Regen Med.

[46] Guo YZ, Li LH, Gao J, Chen XB, Sang QH (2017). miR-214 suppresses the osteogenic differentiation of bone marrow-derived mesenchymal stem cells and these effects are mediated through the inhibition of the JNK and p38 pathways. Int J Mol Med.

[47] Houten SM, Wanders RJA, Ranea-Robles P (2020). Metabolic interactions between peroxisomes and mitochondria with a special focus on acylcarnitine metabolism. Biochim Biophys Acta Mol Basis Dis.

