# Supplementary material for: MicroRNA analysis reveals the role of miR-214 in duck adipocyte differentiation
Source: Anim Biosci. 2022 Jan 21;35(9):1327–39. doi: 10.5713/ab.21.0441 (PMC9449393; doi:10.5713/ab.21.0441)
Supplement: Supplementary file 1 [file ab-21-0441-suppl1.pdf]

Supplementary Table S1 Overview of sequencing data for small RNA.

Sample	Total reads	Clean reads	Clean Ratio（%)	Total sRNA	Mapped sRNA	Mapped ratio (%)	Q20
CVC1	12244755	11614612	0.948537721	11388189	10839223	95.18%	95.62%
CVC2	10749053	10227841	0.951510891	9990793	9450046	94.59%	95.73%
CVC3	11688816	11095066	0.949203581	10776796	10223775	94.87%	95.64%
CVT1	11178383	10650292	0.952757836	10322580	9796856	94.91%	95.70%
CVT2	11706221	11006776	0.940250146	10722896	10252423	95.61%	95.69%
CVT3	11456503	10615983	0.926633808	10331661	9873769	95.57%	95.67%

Supplementary Table S2 Overview of known miRNA comparison in each sample.

Types	Total	CVC1	CVC2	CVC3	CVT1	CVT2	CVT3
Mapped mature	281	230	240	240	226	235	224
Mapped hairpin	210	180	183	189	182	183	180
Mapped uniq sRNA	24705	2018	1995	2089	1795	1773	1719
Mapped total sRNA	76730418	7173999	6051763	6583678	6621922	7142699	6651510

Supplementary Table S3 Overview of differential expression miRNAs.

sRNA	CVT_readcount	CVC_readcount	Fold change	log2FoldChange	P value	Q value
let-7k-5p	2306.8837	3087.2564	0.7472278	-0.4101	0.0037726	0.020216
miR-101-3p	33703.05	38964.801	0.8649614	-0.20818	0.0024886	0.013858
miR-103-3p	20318.713	31917.772	0.6365956	-0.62965	0.0001773	0.0011987
miR-106-5p	3005.6197	4508.7727	0.6666159	-0.56415	0.0010777	0.0066537
miR-10a-5p	2449.342	3921.6463	0.6245699	-0.64886	0.0007171	0.0045259
miR-10b-3p	284.10497	43.325478	6.5574573	2.6093	2.73E-48	3.88E-46
miR-10b-5p	36180.679	5901.1203	6.1311543	2.5598	7.33E-80	2.08E-77
