# Supplementary material for: MicroRNA analysis reveals the role of miR-214 in duck adipocyte differentiation
Source: Anim Biosci. 2022 Jan 21;35(9):1327–39. doi: 10.5713/ab.21.0441 (PMC9449393; doi:10.5713/ab.21.0441)
Supplement: Supplementary file 3 [file ab-21-0441-suppl3.pdf]

Supplementary Table S1 Overview of sequencing data for small RNA.

Sample	Total reads	Clean reads	Clean Ratio（%)	Total sRNA	Mapped sRNA	Mapped ratio (%)	Q20
CVC1	12244755	11614612	0.948537721	11388189	10839223	95.18%	95.62%
CVC2	10749053	10227841	0.951510891	9990793	9450046	94.59%	95.73%
CVC3	11688816	11095066	0.949203581	10776796	10223775	94.87%	95.64%
CVT1	11178383	10650292	0.952757836	10322580	9796856	94.91%	95.70%
CVT2	11706221	11006776	0.940250146	10722896	10252423	95.61%	95.69%
CVT3	11456503	10615983	0.926633808	10331661	9873769	95.57%	95.67%

Supplementary Table S2 Overview of known miRNA comparison in each sample.

Types	Total	CVC1	CVC2	CVC3	CVT1	CVT2	CVT3
Mapped mature	281	230	240	240	226	235	224
Mapped hairpin	210	180	183	189	182	183	180
Mapped uniq sRNA	24705	2018	1995	2089	1795	1773	1719
Mapped total sRNA	76730418	7173999	6051763	6583678	6621922	7142699	6651510

Supplementary Table S3 Overview of differential expression miRNAs.

sRNA	CVT_readcount	CVC_readcount	Fold change	log2FoldChange	P value	Q value
let-7k-5p	2306.8837	3087.2564	0.7472278	-0.4101	0.0037726	0.020216
miR-101-3p	33703.05	38964.801	0.8649614	-0.20818	0.0024886	0.013858
miR-103-3p	20318.713	31917.772	0.6365956	-0.62965	0.0001773	0.0011987
miR-106-5p	3005.6197	4508.7727	0.6666159	-0.56415	0.0010777	0.0066537
miR-10a-5p	2449.342	3921.6463	0.6245699	-0.64886	0.0007171	0.0045259
miR-10b-3p	284.10497	43.325478	6.5574573	2.6093	2.73E-48	3.88E-46
miR-10b-5p	36180.679	5901.1203	6.1311543	2.5598	7.33E-80	2.08E-77

miR-122-5p	53.599376	15.825499	3.3868997	1.4992	6.24E-06	0.0000506
miR-128-3p	16002.497	10921.052	1.4652889	0.53715	0.0002488	0.0016435
miR-1306-3p	552.77889	303.72615	1.8199911	0.84406	1.60E-10	2.84E-09
miR-130b-3p	24590.073	16268.914	1.511476	0.59258	1.61E-17	5.71E-16
miR-140-5p	7714.9104	3720.9099	2.0733935	1.0278	1.53E-13	3.61E-12
miR-1416-5p	44.120014	93.070995	0.4740469	-1.022	5.14E-07	5.84E-06
miR-142-3p	526.22263	762.86341	0.6897993	-0.52968	4.56E-06	0.0000392
miR-142-5p	218.90073	316.5405	0.691541	-0.51871	0.0000205	0.0001573
miR-1434	449.21893	104.57208	4.2957826	1.6911	4.47E-06	0.0000392
miR-146b-3p	199.98432	377.11628	0.5302988	-0.86533	0.0000494	0.000369
miR-146b-5p	186585.21	151338.87	1.2328968	0.30074	9.08E-07	9.92E-06
miR-146c-5p	102007.81	57460.902	1.775256	0.81219	1.93E-10	3.03E-09
miR-148a-3p	4691030.6	2188305.3	2.1436819	1.0837	8.25E-22	4.68E-20
miR-15c-5p	1132.4837	779.64139	1.45257	0.53383	6.49E-12	1.23E-10
miR-16c-5p	10673.817	13448.557	0.7936775	-0.33191	4.93E-08	5.83E-07
miR-181b-1-3p	32.026927	84.158178	0.3805563	-1.2616	1.42E-06	0.0000139
miR-181b-5p	3666.638	4843.2501	0.7570615	-0.39924	2.01E-10	3.03E-09
miR-190a-5p	91.752254	48.280607	1.9003956	0.89108	2.69E-06	0.0000247
miR-193a-3p	60.133549	21.273461	2.8266933	1.3869	1.44E-08	1.82E-07
miR-193b-3p	241.43909	583.48661	0.4137869	-1.1736	2.06E-06	0.0000195
miR-199-3p	198075.06	237647.91	0.8334812	-0.26073	0.0014638	0.0086607
miR-19a-3p	740.5782	465.6346	1.5904707	0.63665	0.0018546	0.010749
miR-206	522.13015	1068.3601	0.4887211	-1.0038	2.03E-10	3.03E-09
miR-20b-5p	2945.0823	4454.5545	0.6611396	-0.58983	3.91E-09	5.55E-08

miR-21-3p	531.04983	166.2046	3.1951573	1.6186	1.55E-21	7.32E-20
miR-214	4832.1703	8367.2573	0.5775095	-0.75711	0.0000752	0.0005338
miR-21-5p	2307160.5	1244651.1	1.8536605	0.87499	5.68E-13	1.15E-11
miR-218-5p	67540.279	36435.655	1.8536864	0.87652	2.92E-14	8.28E-13
miR-219a	233.67667	117.77999	1.9840099	0.94247	1.02E-06	0.0000104
miR-219b	233.67667	117.77999	1.9840099	0.94247	1.02E-06	0.0000104
miR-223	144.07606	300.44363	0.4795444	-1.0154	1.45E-08	1.82E-07
miR-22-3p	12408.967	7450.6878	1.6654793	0.72732	3.82E-13	8.35E-12
miR-27b-3p	6817.837	10068.769	0.6771272	-0.54055	0.002723	0.014872
miR-27b-5p	61.735284	101.45489	0.6084998	-0.66792	0.0059912	0.030384
miR-30a-3p	549.80599	412.91733	1.3315159	0.4045	0.0003819	0.0024647
miR-30a-5p	20570.111	16912.805	1.2162448	0.28108	0.00002	0.0001573
miR-30c-1-3p	519.0533	714.00408	0.7269612	-0.45471	4.82E-06	0.0000403
miR-30d	40581.7	31244.429	1.2988459	0.37608	8.37E-14	2.16E-12
miR-31-5p	31.590987	264.0559	0.1196375	-2.6478	4.62E-17	1.46E-15
miR-32-3p	288.88417	137.06282	2.1076772	1.0603	3.56E-19	1.45E-17
miR-32-5p	1417.7639	1078.4529	1.3146275	0.38342	0.01023	0.049315
miR-455-5p	2921.3158	1121.4136	2.6050297	1.3591	2.10E-31	1.49E-29
miR-458a-3p	17.903463	33.417551	0.5357503	-0.80403	0.0046015	0.02376
miR-458b-5p	17.903463	33.417551	0.5357503	-0.80403	0.0046015	0.02376
miR-460a-5p	169.17271	332.26462	0.5091505	-0.88304	0.0012019	0.0072623
miR-460b-5p	163.37092	285.2547	0.5727195	-0.78673	1.47E-08	1.82E-07
miR-499-5p	492.16371	89.303807	5.5111167	2.3798	9.03E-47	8.55E-45
miR-99a-5p	90349.955	53232.808	1.6972608	0.73103	0.000094	0.0006511

novel_52	699.57197	458.41335	1.5260724	0.57461	0.0084881	0.042292
novel_74	341.13489	145.92575	2.3377293	1.1048	0.0000594	0.0004324

Supplementary Table S4 Overview of differential expression target genes of DEmiRNAs involved in fatty acid metabolism.

gene_name	CVT_FPKM	CVC_FPKM	log2(foldchange)	P value	Q value
CPT2	189.5	133.7905	0.502222	0.00025	0.001085
ACSL1	52.0587	40.1353	0.37527	0.0046	0.013338
HADHB	76.6106	60.2862	0.345717	0.0085	0.022554
HACD3	55.718	30.5248	0.868164	0.00005	0.000251
ACSBG2	26.1296	36.565	-0.48478	0.00035	0.001451
ACAA2	517.401	284.958	0.860532	5.00E-05	0.000251
HADH	257.836	320.408	-0.31346	0.01955	0.045673
FASN	124.336	75.4811	0.720059	0.00005	0.000251

Supplementary Table S5 Overview of target sites of related DEmiRNAs and target genes analysed by Miranda.

sRNA	Target gene_name	score	energy	alian length	miRNA length	gene 3'utr length	position
miR-16c-5p	*ACAA2*	155	-16	16	22	569	355
miR-20b-5p	*CPT2*	150	-15.36	9	23	1537	1351
miR-106-5p	*CPT2*	148	-13.42	11	22	1537	1352
miR-27b-3p	*CPT2*	149	-20.7	18	21	1537	352
miR-16c-5p	*CPT2*	148	-14.87	19	22	1537	812
miR-214	*CPT2*	154	-18.37	17	21	1537	758
miR-214	*CPT2*	140	-15.78	7	21	1537	899
miR-20b-5p	*ACSL1*	155	-13.35	22	23	1456	833
miR-106-5p	*ACSL1*	155	-12.89	21	22	1456	834
miR-1416-5p	*ACSL1*	153	-18.75	14	21	1456	1289
