# Supplementary material for: MicroRNA analysis reveals the role of miR-214 in duck adipocyte differentiation
Source: Anim Biosci. 2022 Jan 21;35(9):1327–39. doi: 10.5713/ab.21.0441 (PMC9449393; doi:10.5713/ab.21.0441)
Supplement: Supplementary file 4 [file ab-21-0441-suppl4.pdf]

novel_52	699.57197	458.41335	1.5260724	0.57461	0.0084881	0.042292
novel_74	341.13489	145.92575	2.3377293	1.1048	0.0000594	0.0004324

Supplementary Table S4 Overview of differential expression target genes of DEmiRNAs involved in fatty acid metabolism.

gene_name	CVT_FPKM	CVC_FPKM	log2(foldchange)	P value	Q value
CPT2	189.5	133.7905	0.502222	0.00025	0.001085
ACSL1	52.0587	40.1353	0.37527	0.0046	0.013338
HADHB	76.6106	60.2862	0.345717	0.0085	0.022554
HACD3	55.718	30.5248	0.868164	0.00005	0.000251
ACSBG2	26.1296	36.565	-0.48478	0.00035	0.001451
ACAA2	517.401	284.958	0.860532	5.00E-05	0.000251
HADH	257.836	320.408	-0.31346	0.01955	0.045673
FASN	124.336	75.4811	0.720059	0.00005	0.000251

Supplementary Table S5 Overview of target sites of related DEmiRNAs and target genes analysed by Miranda.

sRNA	Target gene_name	score	energy	alian length	miRNA length	gene 3'utr length	position
miR-16c-5p	*ACAA2*	155	-16	16	22	569	355
miR-20b-5p	*CPT2*	150	-15.36	9	23	1537	1351
miR-106-5p	*CPT2*	148	-13.42	11	22	1537	1352
miR-27b-3p	*CPT2*	149	-20.7	18	21	1537	352
miR-16c-5p	*CPT2*	148	-14.87	19	22	1537	812
miR-214	*CPT2*	154	-18.37	17	21	1537	758
miR-214	*CPT2*	140	-15.78	7	21	1537	899
miR-20b-5p	*ACSL1*	155	-13.35	22	23	1456	833
miR-106-5p	*ACSL1*	155	-12.89	21	22	1456	834
miR-1416-5p	*ACSL1*	153	-18.75	14	21	1456	1289
