# Supplementary material for: MicroRNA analysis reveals the role of miR-214 in duck adipocyte differentiation
Source: Anim Biosci. 2022 Jan 21;35(9):1327–39. doi: 10.5713/ab.21.0441 (PMC9449393; doi:10.5713/ab.21.0441)
Supplement: Supplementary file 6 [file ab-21-0441-suppl6.pdf]

```
Read Sequence:CPT2 (1537 nt)
=-=-=-=-=-=-=-=-=-=-=-=-=-=-=-=-=-=-=-=-=-=-=-=-=-=-=-=-=-=-=-=
Performing Scan: gga-miR-214 vs CPT2
=-=-=-=-=-=-=-=-=-=-=-=-=-=-=-=-=-=-=-=-=-=-=-=-=-=-=-=-=-=-=-=

   Forward: Score: 154.000000  Q:2 to 19  R:758 to 778 Align Len (17) (70.59%) (76.47%)

   Query:    3' gacGGACAGACACGGACGACa 5'
                   |:| ||   ||||||||
   Ref:      5' ataCTTTTCAAAGCCTGCTGc 3'

   Energy:  -18.370001 kCal/Mol

Scores for this hit:
>gga-miR-214    CPT2    154.00  -18.37  2 19    758 778 17  70.59%  76.47%


   Forward: Score: 140.000000  Q:2 to 9  R:899 to 919 Align Len (7) (100.00%) (100.00%)

   Query:    3' gacggacagacacGGACGACa 5'
                             |||||||
   Ref:      5' ttccaatattcctCCTGCTGt 3'

   Energy:  -15.780000 kCal/Mol

Scores for this hit:
>gga-miR-214    CPT2    140.00  -15.78  2 9 899 919 7   100.00% 100.00%

Score for this Scan:
Seq1,Seq2,Tot Score,Tot Energy,Max Score,Max Energy,Strand,Len1,Len2,Positions
>>gga-miR-214   CPT2    294.00  -34.15  154.00  -18.37  871745  21  1537     758 899
Complete
```

Supplementary Figure S1. The target relationship of miR-214 and CPT2 analyzed by miRanda.
