# Supplementary material for: MicroRNA analysis reveals the role of miR-214 in duck adipocyte differentiation
Source: Anim Biosci. 2022 Jan 21;35(9):1327–39. doi: 10.5713/ab.21.0441 (PMC9449393; doi:10.5713/ab.21.0441)
Supplement: Supplementary file 7 [file ab-21-0441-suppl7.pdf]

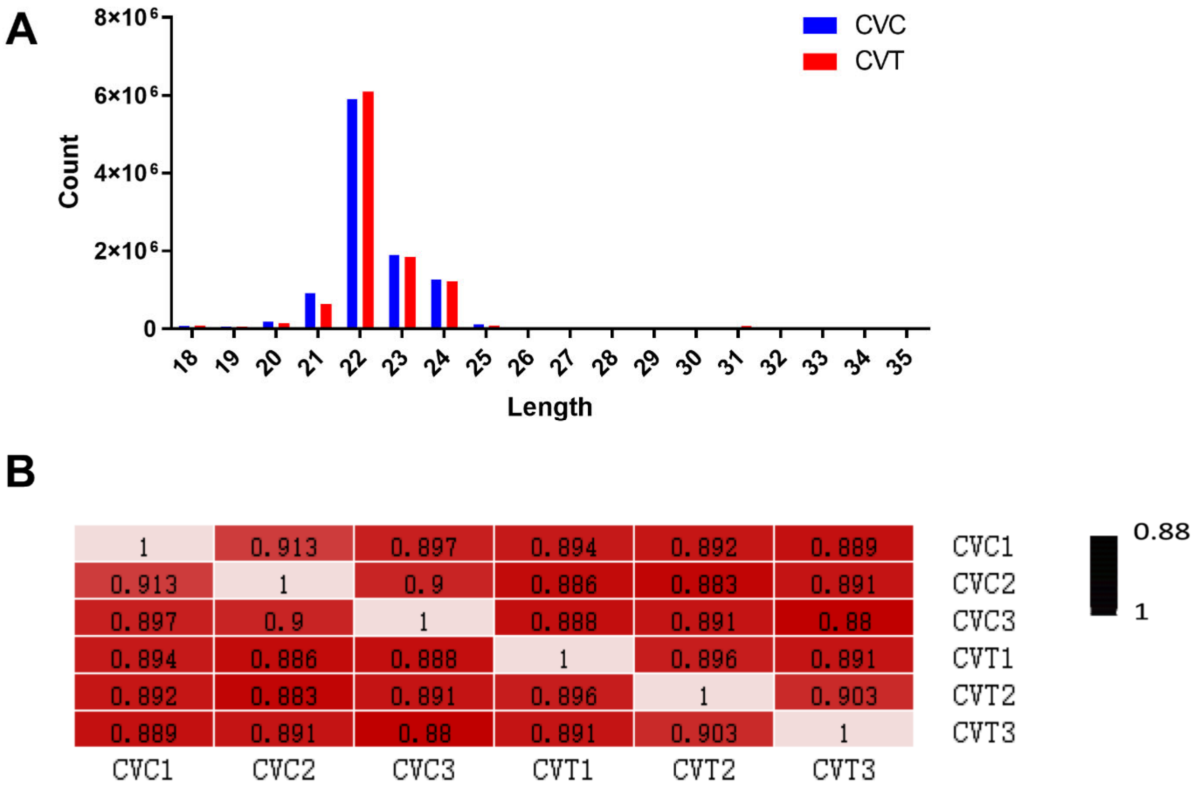

Supplementary Figure 2S. (A) Distributions of the length and abundance of small RNA sequences. (B) Correlation between samples in the number of small RNA sequences detected.
