# Supplementary material for: MicroRNA analysis reveals the role of miR-214 in duck adipocyte differentiation
Source: Anim Biosci. 2022 Jan 21;35(9):1327–39. doi: 10.5713/ab.21.0441 (PMC9449393; doi:10.5713/ab.21.0441)
Supplement: Supplementary file 8 [file ab-21-0441-suppl8.pdf]

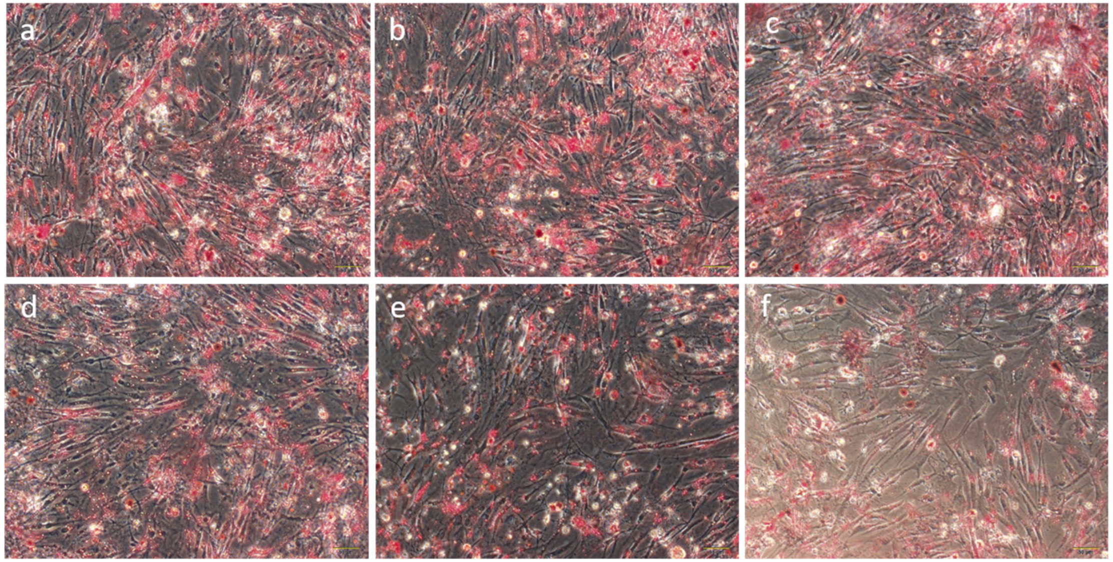


**Supplementary Figure 3S.** (a, b, c) Oil Red O staining by duck adipocytes transfected with miR-214 mimics for 48 h and differentiation for 4 d. (d, e, f) Oil Red O staining by duck adipocytes transfected with miR-NC for 48 h and differentiation for 4 d. n = 3 independent experiments.
